# The Problems of Burial Insurance

**Published:** 1897-08-07

**Authors:** 


### Modern Sociology.

### THE PROBLEMS OF BURIAL INSURANCE.

Among the many marks of moral progress which may be recorded of the Victorian Era, perhaps few are more striking than the attention now bestowed by the Legislature and society generally on the care and education of children, especially those belonging to the lower classes. In the last centuries the offspring of parents, either unable or unwilling to give them due nurture and training, were left to struggle up into mature life as best they could, little or nothing being done to secure their well-being morally or physically in any appreciable degree. It is very different at the present time, and the efforts made on all sides for the benefit of the rising generation are too well known to require any description in these pages; but there is one danger to child-life, connected with what is supposed to be a very beneficent institution, which should be constantly kept in view by those who have the interest of the children at heart. At first sight the system of insuring lives for the expenses of burial in the event of death seems to be a useful and convenient arrangement for persons who might be able to pay a very small sum regularly, but on whom the sudden heavy cost of a funeral would fall as a disastrous calamity. Nevertheless, under this apparently harmless agency there lurks a most serious and subtle evil, of which we have ourselves witnessed the baneful effects in many recent cases.

The system certainly appears quite unobjectionable, and even meritorious, when a respectable working man—simply desiring to protect himself and his wife from future responsibilities they might not be able to meet—decides to pay the burial insurance fees for himself and all the members of his family, as among his numerous sons and daughters it is very possible that one or more may precede him to the grave. In any case, it is a safeguard for the children themselves against the expenses which must sooner or later fall upon them in providing for the interment of their parents. The case is, however, widely different when a woman with an illegitimate child—perhaps only an infant newly-born—hastens to pay the small insurance premium, and that when the child is thoroughly healthy and likely to live. There is something very sinister and unnatural in a mother contemplating the death of an infant, which so many of her sex would look upon as a gift from heaven, or at least a greatly-prized treasure; yet it is well-known that there exists a widespread system by which these burial insurances may be utilised for purposes of gain, and the means employed can only be rightly described as deliberate child murder, carefully planned and accomplished. There are various different methods by which sums of money can, after this manner, be gained from the insurance agencies. For instance, if a small weekly or monthly premium is to be paid for an infant who dies after the first instalment has been remitted, the full burial money, greatly exceeding that amount, is at once received; and there are besides various nefarious practises by which it is possible to avoid using the money at all for the funeral. Infants have been buried secretly at dead of night in ground never likely to be searched, or sent in parcels by railway to destinations where they were totally unknown. As the burial money is in some cases paid immediately on the death being certified, the subsequent disposal of the body may not be inquired into by the agents. Another method possible to persons who can be rated as paupers is as follows: If a child who has been insured dies it is not difficult to conceal the fact of its insurance from the parochial authorities, and to plead poverty sufficiently to have it buried by the parish, so that all expense is avoided, and the insurance money secured intact. The same end can, however, be attained by expedients of deeper dye than mere fraud. We have spoken in a previous paper of the prevalence of infanticide by the parents of illegitimate children, and the victims of the burial insurance system are most frequently taken from that unhappy class; but a very large proportion can be found also in households where a stepmother or stepfather rules over a family of children to whom they stand in that ominous relation. In this connection we had the satisfaction of seeing a woman of that class in prison under the following circumstances: She had married a widower who had one little boy by his first wife; three children were the offspring of the second marriage. The woman had insured this first child, and this one alone out of all the family, in the burial agency. It became known to the police that she was deliberatively starving him to death, and she was sentenced to a term of imprisonment she richly deserved. The discovery of the truth in this case alone distinguishes it from innumerable practices of the same kind which are secretly carried on in connection with the burial agency. Illegitimate children who have no protector but the unwedded mother, earnestly desirous of being freed from an unwelcome burden, are often disposed of by much more summary measures; here is a typical case, known in all its details to ourselves: A girl who had led a disreputable life on the streets from an early age, under the auspices of a most evil-minded mother of like tendencies, gave birth to a child, which she immediately insured for burial; the infant, however, was perfectly healthy—likely to live and grow to maturity. After a time, when she found the child still alive and the burial money unclaimed, she and her mother took counsel together, with the result that they deftly overturned a pan of boiling water on the unhappy infant, who speedily succumbed to the cruel injuries it sustained, and the verdict of accidental death having been given at the inquest, the burial money was secured—greatly exceeding the small premium that had been paid. We could multiply instances of the crafty murder of bastard children as well as others in this connection did our space permit, but we have sufficiently shown that the matter really constitutes a very critical problem. We have ourselves exemplified the righteous and beneficent working of the system when used by honest and respectable persons; while the specimen cases we have adduced prove that it opens the door to endless possibilities of child murder.

